# Paraquat-induced systemic inflammation and increased oxidative markers in rats improved by *Zataria multiflora* extract and carvacrol

**Published:** 2020

**Authors:** Fatemeh Amin, Ali Roohbakhsh, Arghavan Memarzia, Hamid Reza Kazerani, Mohammad Hossein Boskabady

**Affiliations:** 1 *Department of Physiology, Faculty of Veterinary Medicine, Ferdowsi University of Mashhad, Mashhad, Iran*; 2 *Pharmaceutical Research Center, Pharmaceutical Technology Institute, Mashhad University of Medical Sciences, Mashhad, Iran*; 3 *Department of Physiology, School of Medicine, Mashhad University of Medical Sciences, Mashhad, Iran*; 4 *Neurogenic Inflammation Research Center, Mashhad University of Medical Sciences, Mashhad, Iran*

**Keywords:** Paraquat, Zataria multiflora, Carvacrol, Oxidative biomarkers, Inflammation

## Abstract

**Objective:**

Paraquat (PQ) is a herbicide which induces oxidative stress and inflammation. Anti-inflammatory and anti-oxidant effects were shown for* Zataria multiflora* (Z*. multiflora*) and carvacrol previously. The effects of Z*. multiflora* hydroalcoholic extract and carvacrol on systemic inflammation and oxidative stress induced by inhaled PQ were examined in this study.

**Materials and Methods:**

Six groups of male rats used in this study were as follows: control group exposed to normal saline aerosol, one group exposed to PQ 54 mg/m^3^ aerosol, animals exposed to PQ 54 mg/m^3^ and treated with Z*. multiflora* (200 and 800 mg/kg/day) or carvacrol (20 and 80 mg/kg/day) for 16 days after the end of exposure to PQ. Exposure to PQ was performed 8 times, every other day, each time for 30 min. After the end of the treatment period, different variables were measured.

**Results:**

Significant increases in nitrite (NO_2)_, malondialdehyde (MDA) and interleukin (IL)-6 serum levels but significant reduction of interferon-gamma (IFN-γ) serum levels as well as IFN-γ/IL-6 ratio were observed in PQ-exposed compared to control group (p<0.01 for MDA and IL-6, p<0.001 for other cases). Treatment with both doses of the extract and carvacrol specially at high dose, reduced MDA, NO_2_, and IL-6 but increased IFN-γ and IFN-γ/IL-6 ratio compared to un-treated PQ exposed group (p<0.05 to p<0.001).

**Conclusion:**

Treatment with *Z. multiflora* and carvacrol improved systemic inflammation oxidative biomarkers induced by inhaled PQ which may indicate therapeutic potential of the plant and its constituent, carvacrol in systemic inflammation and oxidative biomarkers induced by inhaled PQ.

## Introduction

Over the last decades, agrochemicals have been widely used to eliminate weeds and increase the yield of agricultural products. However, these compounds might impose serious risks to human health (Dhananjayan and Ravichandran, 2018 ▶). Paraquat (PQ) -1, 1´-dimethyl-4, 4´-bipyridinium dichloride- is an effective and widely used herbicide (Dinis-Oliveira et al., 2008 ▶) that can potentially induce serious toxicity in humans (Kim et al., 2008 ▶). The PQ poisoning is classified as mild, moderate, severe on the basis of the doses used (de Maglia Botella and Belenguer, 2000 ▶). The definitive mechanism of toxicity of PQ has not been described, but one of the most important mechanisms of PQ toxicity is inducing of oxidative stress and inflammation (Liu et al., 2019 ▶). Finally, PQ exposure can lead to disruption of NADPH, which is needed for normal cell function (Morán et al., 2010 ▶).

*Zataria multiflora* (*Z. multiflora*) is a perennial plant with small, elliptical, and greenish-grey leaves and a strong and pleasant aroma, growing in Iran, Pakistan and Afghanistan (Boskabady and Gholami Mhtaj, 2014 ▶). The anti-inflammatory effects of the plant and its components carvacrol were shown by reduction of total and differential white blood cell counts as well as inflammatory mediators in an animal model of asthma and chronic obstructive pulmonary diseases (COPD) (Boskabady and Mahtaj, 2015 ▶). *Z. multiflora* and carvacrol also showed effects on oxidative damage, inflammation and reduced oxidative stress by scavenging free radicals; also, they exerted protective effects on serum levels of nitric oxide (NO_2)_ and malondialdehyde (MDA) (Ahmadipour et al., 2015 ▶). Thymol and carvacrol are the two main compounds of *Z. multiflora* and carvacrol may participate in the anti-inflammatory effects of the plant by inhibiting inflammatory edema and leukocyte migration (Fachini-Queiroz et al., 2012 ▶).

According to the anti‐inflammatory and antioxidant effects of *Z. multiflora* and its constituent, carvacrol, their effects on systemic inflammation and oxidative biomarkers induced by inhaled PQ in rats, were examined in the present study.

## Materials and Methods

**Animal and groups**

Male Wistar rats (200-250 g) were used in the present study and maintained in the Animal house, School of Medicine, Mashhad University of Medical Sciences, Mashhad, Iran in plexiglas cages under standard condition of 12 hr light/dark cycle, at 22±2°C with humidity of 54±2% and they had free access to food and water during the experimental period. The study was approved by the ethics committee of Mashhad University of Medical Sciences for Animal Experiments (No. 961202) Animal experiments were done according to criteria outlined in the Guide for Care and Use of Laboratory Animals (NIH US publication 23-68 revised 1985; http://oacuod.nih.gov/regs/guide/guidex.htm). The animals were randomly assigned into the following six groups (n=5 in each group) (Table 1). 

a) Animals exposed to normal saline aerosol 8 times, every other day, during 16 days (control group).

b) Animals exposed to PQ (Sigma Aldrich Co, China) aerosol (Burleigh-Flayer et al., 1987 ▶) at the dose of 54 mg/m^3^ 8 times, every other day, during 16 days (PQ group). 

c) Animals exposed to PQ and treated with 200 mg/kg/day *Z. multiflora* extract by gavage for 16 days after the end of PQ exposure period (Heydari et al., 2019 ▶). 

d) Animals exposed to PQ and treated with 800 mg/kg/day *Z. multiflora* extract by gavage for 16 days after the end of PQ exposure period (Heydari et al., 2019 ▶).

 e) Animals exposed to PQ and treated with 20 mg/kg/day carvacrol by gavage for 16 days after the end of PQ exposure period (Jalali et al., 2013 ▶).

f) Animals exposed to PQ and treated with 80 mg/kg/day carvacrol by gavage for 16 days after the end of PQ exposure period (Jalali et al., 2013 ▶).

**Table 1:** Six groups of rats were included in the present study, their exposing to saline or paraquate and treatment protocol

**groups**	**Exposed to**	**Treatment with**
**Control**	Salin aerosol	_
**PQ **	PQ aerosol (54 mg/m^3^) for 30 min, 8 times every other day order over 16 days	_
**Z-l **	PQ aerosol (54 mg/m^3^) for 30 min, 8 times every other day order over 16 days	*Z. multiflora* extract low dose (200 mg/kg/day) for 16 days after the end of PQ exposure
**Z-h **	PQ aerosol (54 mg/m^3^) for 30 min, 8 times every other day order over 16 days	*Z. multiflora* extract high dose (800 mg/kg/day) for 16 days after the end of PQ exposure
**Carv-l**	PQ aerosol (54 mg/m^3^) for 30 min, 8 times every other day order over 16 days	Carvacrol low dose (20 mg/kg/day) for 16 days after the end of PQ exposure
**Carv-h**	PQ aerosol (54 mg/m^3^) for 30 min, 8 times every other day order over 16 days	Carvacrol high dose (80 mg/kg/day) for 16 days after the end of PQ exposure

PQ: Paraquat, *Z. multiflora*: *Zataria multiflora*, Z-l: treated group with low dose of *Z. multiflora* extract, Z-h: treated group with high dose of *Z. multiflora* extract, Carv-l: treated group with low dose of carvacrol, Carv-l: treated group with high dose of carvacrol

**Figure 1:**
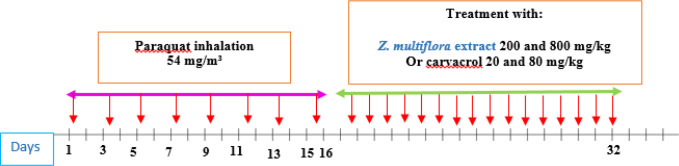
Protocol of exposing animals to inhaled PQ (54 mg/m^3^) and treatment of animals with the extract of *Z. multiflora* and carvacrol.

**Animal exposure to paraquat**

Animal were exposed to PQ (Sigma Aldrich Co, China) aerosol as previously described (Heydari et al., 2019 ▶). Briefly, PQ aerosol was delivered to animal head box dimensions 15×18×30 cm, eight times every other day over 16 days (each time for 30 min/day) at the dose of 54 mg/m^3^ (Burleigh-Flayer and Alarie, 1987 ▶).

**Plant and extract**

The collection of the plant (Herbarium No. 35314, FUMH, identified by Mr Joharchi) and preparation of the extract were done as previously described (Heydari et al., 2019 ▶).

The hydroalcoholic extract and carvacrol were daily gavaged after the end of PQ exposure for 16 days. In control group, saline was used instead of the extract and carvacrol (Figure 1).

After the end of treatment period (day 17), the rats were anesthetized with an intraperitoneal injection of ketamine (1 mg/kg) and xylazine (50 mg/kg). Then, blood samples were taken from the heart and centrifuged and serum was separated and stored at -20°C for oxidant, anti-oxidant biomarkers and cytokine measurement. Total NO_2_ and MDA concentrations were measured as described previously (Saadat et al., 2019 ▶). 

**Cytokines measurement**

Levels of interleukin (IL-6 ) and interferon-gamma (IFN-γ) in serum were measured using specific enzyme-linked immunosorbent assay (ELISA) kits (Karmania Pars Gen, Kerman, Iran) according to the manufacturer instructions as previously described (Shakeri and Boskabady, 2017 ▶). IFN-γ/IL-6 ratio was also calculated.

**Statistical analysis**

Data of different groups was compared using the one-way analysis of variance (ANOVA) followed by Tukey’s multiple comparison test and results are shown as mean±SEM. Values of p<0.05 were considered statistically significant.

## Results

**The effects of paraquat**

**Figure 2:**
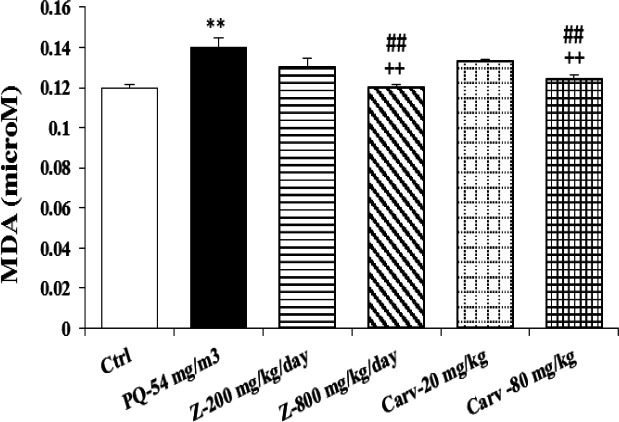
Serum level of malondialdehyde (MDA) in different studied groups. The results are expressed as mean±SEM (n=5 in each group). **p<0.01 compared to the control group. ++p<0.01 compared to the PQ group, ## p<0.01 compared to low dose of *Z. multiflora *and carvacrol*,* Comparisons between different groups were made using one-way ANOVA followed by Tukey’s multiple comparison test. Ctrl, control group, PQ-54 mg/m^3^, group exposed to paraquat aerosol at the dose of 54 mg/m^3^, Carv-20 mg/kg/day, Carv-80 mg/kg/day and Z- 200 mg/kg/day, Z-800 mg/kg/day, groups exposed to PQ-54 mg/m^3^ and treated with 20 and 80 mg/kg/day carvacrol or 200 and 800 mg/kg/day *Zataria multiflora*, respectively

**Figure 3:**
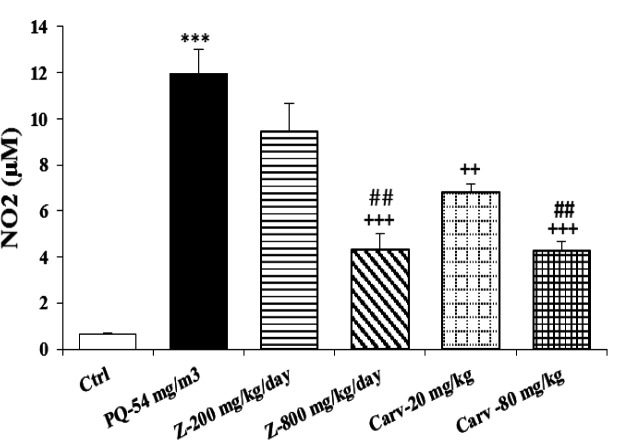
Serum level of nitrite (NO_2_) in different studied groups. The results are expressed as mean±SEM (n=5 in each group). ***p<0.001 compared to the control group. ++p<0.01 and +++p<0.001 compared to PQ group. ## p<0.01 compared to low dose of *Z. multiflora *and carvacrol. Comparisons between different groups were made using one-way ANOVA followed by Tukey’s multiple comparison test. Ctrl, control group, PQ-54 mg/m^3^, group exposed to paraquat aerosol at the dose of 54 mg/m^3^, Carv-20 mg/kg/day, Carv-80 mg/kg/day and Z- 200 mg/kg/day, Z-800 mg/kg/day, groups exposed to PQ-54 mg/m^3^ and treated with 20 and 80 mg/kg/day carvacrol and 200 and 800 mg/kg/day *Zataria multiflora*, respectively

**Figure 4:**
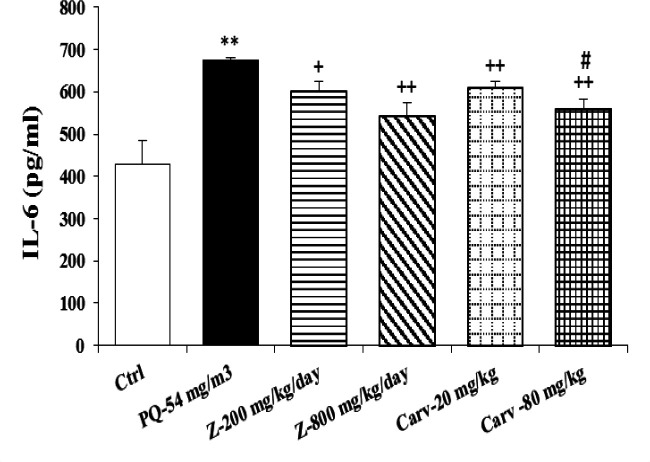
Serum level of interlockin-6 (IL-6) in different studied groups. The results are expressed as mean±SEM (n=5 in each group). **p<0.01 compared to the control group. +p<0.05 and ++p<0.01 compared to the PQ group. #p<0.05 compared to low dose of carvacrol. Comparisons between different groups were made using one-way ANOVA followed by Tukey’s multiple comparison test. Ctrl, control group, PQ-54 mg/m^3^, group exposed to paraquat aerosol at the dose of 54 mg/m^3^, Carv-20 mg/kg/day, Carv-80 mg/kg/day and Z- 200 mg/kg/day, Z-800 mg/kg/day, groups exposed to PQ-54 mg/m^3^ and treated with 20 and 80 mg/kg/day carvacrol and 200 and 800 mg/kg/day *Zataria multiflora*, respectively

**Figure 5:**
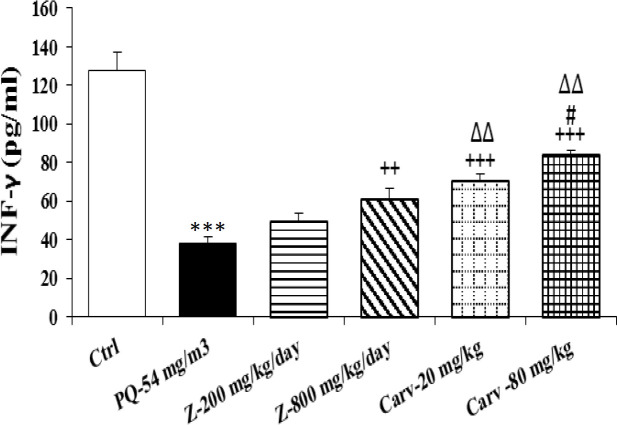
Serum level of interferon gama (INF-γ) in different studied groups. The results are expressed as mean±SEM (n=5 in each group). ***p<0.001 compared to the control group. ++p<0.01 and +++p<0.001 compared to the PQ group*,* ##p<0.01 compared to low dose of *Z. multiflora *and carvacrol*,* ∆∆p<0.01 compared to the effect of *Z. multiflora*. Comparisons between different groups were made using one-way ANOVA followed by Tukey’s multiple comparison test. Ctrl, control group, PQ-54 mg/m^3^, group exposed to paraquat aerosol at the dose of 54 mg/m^3^, Carv-20 mg/kg/day, Carv-80 mg/kg/day and Z- 200 mg/kg/day, Z-800 mg/kg/day, groups exposed to PQ-54 mg/m^3^ and treated with 20 and 80 mg/kg/day carvacrol and 200 and 800 mg/kg/day *Zataria multiflora*, respectively

**Figure 6:**
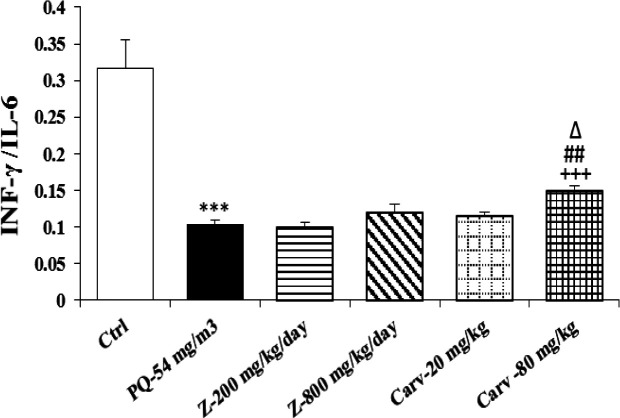
IFN-γ/IL-4 ratio in different studied groups. The results are expressed as mean± SEM (n=5 in each group). ***p<0.001 compared to the control group, +++p<0.001 compared to the PQ group*,* ##p<0.01 compared to low dose of carvacrol*,* ∆p<0.05 compared to the effect of *Z. multiflora*. Comparisons between different groups were made using one-way ANOVA followed by Tukey’s multiple comparison test. Ctrl, control group, PQ-54 mg/m^3^, group exposed to paraquat aerosol at the dose of 54 mg/m^3^, Carv-20 mg/kg/day, Carv-80 mg/kg/day and Z- 200 mg/kg/day, Z-800 mg/kg/day, groups exposed to PQ-54 mg/m^3^ and treated with 20 and 80 mg/kg/day carvacrol and 200 and 800 mg/kg/day *Zataria multiflora*, respectively

Exposure of rats to inhaled PQ (54 mg/m^3^) caused significant increases in MDA and NO_2_ serum levels (p<0.01 and p<0.001 for MDA and NO_2_, respectively), (Figures 2 and 3). The serum level of IL-6 was also increased due to PQ exposure (p<0.01; Figure 4) but INF-γ level and IFN-γ/IL-6 ratio were decreased compared to the control group (p<0.001 for both cases; Figures 5 and 6).

**The effects of** ***Z. multiflora*** **extract treatment**

Treatment with high dose of *Z. multiflora *(800 mg/kg/day) significantly decreased serum levels of MDA and NO_2_ (p<0.01 and p<0.001 for MDA and NO_2_, respectively; Figures 2 and 3). 

Serum level of IL-6 was significantly decreased due to treatment with both doses of *Z. multiflora* (p<0.05 and p<0.01 for low and high doses, respectively; Figure 4). However, only treatment with high dose of the extract significantly increased serum INF-γ compared to PQ-exposed group (p<0.01; Figure 5).

Treatment with high dose of *Z. multiflora *(800 mg/kg/day) improved MDA and NO_2_ significantly higher than low dose of the extract (p<0.01 for both cases; Figures 2 and 3).

**The effects of carvacrol treatment**

Serum level of MDA was significantly decreased due to treatment with high dose of carvacrol (p<0.01) but both doses (20 and 80 mg/kg/day) significantly decreased serum concentration of NO_2_ (p<0.01 and p<0.001 for low and high doses, respectively; Figures 2 and 3). Serum level of IL-6 was significantly decreased in groups treated with both doses of carvacrol (p<0.01 for both doses) but increased the level of IFN-γ (p<0.001 for both doses; Figures 4 and 5). However, IFN-γ/IL-6 ratio was significantly increased in the group treated with high dose of carvacrol compared to PQ group (p<0.001; Figures 2-6).

The effects of high dose of carvacrol (80 mg/kg/day) on MDA, NO_2_, IL-6 and INF-γ levels as well as IFN-γ/IL-6 ratio were significantly higher than its low dose (20 mg/kg/day) (p<0.05 to p<0.01; Figures 2-6).

**Comparison between** ***Z. multiflora*** **and carvacrol effects**

The effects of both doses of carvacrol on IFN-γ level (p<0.01 for both doses) and the effect of its high dose on IFN-γ/IL-6 ratio (p<0.05) were significantly higher than the corresponding doses of *Z. multiflora *(Figures 5 and 6). However, there was no significant difference between the effect of carvacrol and that of the extract on MDA, NO_2_ and IL-6 levels (Figures 2-4).

## Discussion

In the present study, effects of *Z. multiflora *extract and carvacrol on oxidant markers (MDA and NO_2_), cytokine levels of IL-6, IFN-γ in the serum, and IFN-γ/IL-6 ratio as an indicator of Th1/Th2 balance, were investigated in rats exposed to inhaled PQ. The results showed that serum levels of MDA and NO_2_ were significantly increased in rats exposed to 54 mg/m^3^ aerosol of PQ.

Previous studies showed induction of oxidative biomarkers in experimental models of PQ poisoning (Pourgholamhossein et al., 2016 ▶) as well as a positive relation between enhanced levels of MDA and NO_2_ and PQ administration doses. Increased levels of MDA and NO_2_ and decreased SOD, CAT and thiol levels were also shown due to PQ toxicity (Keeling and Smith, 1982 ▶) which confirm the findings of the current study.

Treatment with *Z. multiflora* extract and carvacrol decreased oxidative biomarkers in animals exposed to PQ. The effects of higher dose of the extract and carvacrol were more potent than their low doses which showed a concentration-dependent effect of the plant and its constituent carvacrol on oxidative biomarkers. 

Several studies showed the effects of *Z. multiflora* extract on oxidant stress, which confirm the results of the present study (Khazdair et al., 2018 ▶). *Z. multiflora* extract decreased cisplatin-induced oxidative stress in the liver by improving SOD, CAT, and GSH-Px and glutathione level (Ahmadipour et al., 2015 ▶; Boskabady and Gholami Mhtaj, 2014 ▶) and decreased serum level of MDA in animal models of COPD (Ahmadipour et al., 2015 ▶; Boskabady and Gholami Mhtaj, 2014 ▶). Heydari et al. also showed that impairment of learning and memory as well as lung oxidative stress induced by inhaled PQ, were improved by *Z. multiflora* (Heydari et al., 2019 ▶).

*Z. multiflora* and carvacrol showed preventive effect on lung oxidative stress by increasing thiol content of broncho-alveolar lavage fluid in a guinea pig model of COPD (Boskabady and Mahtaj, 2015 ▶). The antioxidant potential of methanol extracts and essential oil from *Z. multiflora* using diphenyl-1-picrylhydrazyl (DPPH) and ammonium thiocyanate showed reduction of DPPH which was more potent for the essential oil (Sharififar et al., 207 ▶). In the DPPH antioxidant assay, carvacrol the main constituent of *Z. multiflora*, exhibited a remarkable activity (Saei-Dehkordi et al., 2010 ▶). Carvacrol also reduced oxidative stress against irinotecan hydrochloride -induced intestinal mucositis by reduction of GSH, MDA, and NO_2_ levels (Alvarenga et al., 2016 ▶), showed capacity for scavenging free radicals (Miguel et al., 2009 ▶) and decreased myeloperoxidase activity in gingival tissue of periodontium (Botelho et al., 2009 ▶). All the described studies support the results of the present study, indicating the reduction of systemic oxidative biomarkers induced by inhaled PQ by treatment with *Z. multiflora* and its constituent carvacrol. In fact, changes in other oxidative stress inhibitors also showed improvement of pulmonary injury and systemic inflammation due to PQ poisoning as reflected by enhanced SOD, CAT, thiol levels and decreased NO_2_ and MDA concentrations (Klimek, Schaap, and Kimura, 1982 ▶) which confirm the findings of the current study.

The results of the present study also showed increased serum level of IL-6 but decreased IFN-γ in the serum, and IFN-γ/IL-6 ratio in rats exposed to 54 mg/m^3^ aerosol of PQ.

Infiltration of inflammatory cells into the lung microenvironment by PQ administration (Hemmati et al., 2002 ▶) and increased total white blood cells (WBC) by sub-acute administration of PQ were shown. Total WBC, neutrophils, macrophages, lymphocytes and monocytes counts in BALF were increased 48 hr after administration of a single dose of PQ (Liu et al., 2019 ▶). Increased expression of inflammatory cytokines in the blood of patients exposed to inhaled PQ (Meng et al., 2019 ▶) as well as decreased levels of IL-10 and IFN-γ in the blood (Litteljohn et al., 2009 ▶) were reported which support the findings of the present study regarding the induction of systemic inflammation by inhaled PQ. Increased IL-6 level was observed following PQ treatment of mouse macrophage RAW2647 (Huang et al., 2019 ▶). It was also demonstrated that IFN-γ prevent the PQ-induced neurodegeneration and the accompanying oxidative and inflammation (Mangano et al., 2012 ▶). Therefore, Th1/Th2 balance in PQ-induced inflammation similar to the condition that exists in some other inflammatory disorders such as asthma.

Treatment with *Z. multiflora* extract and carvacrol decreased IL-6 level but increased IFN-γ level and IFN-γ/IL-6 ratio in a concentration-dependent manner, i.e. the effects of higher dose of the extract and carvacrol were greater than their low dose.

Anti-inflammatory effects of *Z. multiflora* and carvacrol were previously shown in several studies. Treatment with *Z. multiflora* extract and carvacrol in spleen cells from ovalbumin-sensitized mice, reduced expression of inflammatory cytokines including IL-4, TGF-β, IL-17, and decreased the expression of anti-inflammatory cytokines INF-γ and FOXP3 (Kianmehr et al., 2019 ▶). Serum levels of NO, NO_2_, phospholipase A2 (PLA2), total protein (TP) and histamine were improved in sensitized guinea pigs treated with *Z. multiflora* extract (Boskabady et al., 2014a). Treatment with the extract of *Z. multiflora* and its constituent, carvacrol also improved total and differential WBC, IgE and eosinophil peroxidase levels as well as lung pathology in an animal model of asthma (Boskabady et al., 2014b). The effects of *Z. multiflora* and carvacrol on lung inflammation in guinea pig model of COPD were shown and they improved total and differential WBC and IL-8 level in broncho-alveolar lavage fluid (Boskabady and Mahtaj, 2015 ▶). Also, *Z. multiflora* and its main constituent, carvacrol, led to reduction of total and differential WBC as well as serum level of IL-8 in an animal model of COPD (Boskabady and Gholami Mhtaj, 2014 ▶).

Carvacrol treatment also improved serum levels of total protein, phospholipase A2 and histamine in an animal model of asthma (Boskabady et al., 2016 ▶). In irinotecan hydrochloride-induced intestinal mucositis, carvacrol showed anti-inflammatory effects by reduction of pro-inflammatory cytokines such as TNF-α and IL-1β (Alvarenga et al., 2016 ▶). These studies confirm the findings of the present study regarding the effect of the plant extract and its constituent on systemic inflammation induced by PQ aerosol.

The results of the current study suggest that carvacrol treatment increased IFN-γ/IL-6 ratio. While IFN-γ produced by t-helper (Th1) and IL-6 by Th2 cells, carvacrol showed stimulatory effect on Th1 and inhibitory effect on Th2 helper cells. In fact, the increment of IFN-γ/IL-6 ratio indicated the increased Th1/Th2 balance which could be of therapeutic value in inflammatory conditions such as asthma and cancer. A previous study showed increased Th1/Th2 balance due to *Z. multiflora* extract treatment in *in vitro* and *in vivo* model of asthma and stimulated human lymphocyte (Boskabady et al., 2013 ▶).

The effects of carvacrol on oxidant markers were equal but on IL-6, IFN-γ and IFN-γ/IL-6 ratio were higher than the effects of the extract. These findings suggest that carvacrol, the main constituent of *Z. multiflora* is perhaps responsible for the observed effects of the plant. However, according to the results of the present study, the possible mechanisms of the extract and carvacrol are their antioxidant and immunomodulatory effects which resulted in anti-inflammatory effects. In fact, antioxidant, immunomodulatory and anti-inflammatory effects of the plant and carvacrol were reported in numerous studies as discussed earlier.

Extract and carvacrol doses were selected based on previous animal studies (Boskabady et al., 2013 ▶; Boskabady et al., 2016 ▶). In addition, clinical studies on asthmatic patients and sulfur mustard exposed veterans showed the absence of side effects of equivalent doses of the *Z. multiflora* extract and carvacrol in humans. In addition, the safety and toxicity of carvacrol were also studied and no side effect was reported for this agent (Ghorani, et al ., 2018 ▶; Khazdair et al., 2018 ▶).

One of the limitation of the current study is the absence of a positive control group. However, in several previous studies, similar effects for the plant and carvacrol and dexamethasone were reported (Bosbabady et al., 2013 ▶; Bosbabady et al., 2014a; Bosbabady et al 2014b; Bosbabady et al 2015 ▶; Bosbabady et al., 2016 ▶; Kianmehr et al., 2019 ▶). Therefore, in this study, a positive group was not evaluated. The other limitation of the study is the absence of examination of antioxidant markers which should be examined in further studies. The effect of *Z. multiflora* and carvacrol on total and differential WBC as well as other inflammatory mediators in animals exposed to inhaled PQ, also should be examined in further studies.

The extract of *Z. multiflora* and carvacrol showed preventive effect on systemic oxidative biomarkers (MDA and NO_2_) and inflammation (cytokines levels) in rats exposed to inhaled PQ. The results also suggest that the effects seen for the extract, are due to its constituent carvacrol.
